# Identification of a lipid metabolism-related gene for cancer immunotherapy

**DOI:** 10.3389/fphar.2023.1186064

**Published:** 2023-05-12

**Authors:** Xin Jiang, Wenqi Du, Ce Shi, Mengjie Kang, Qiuya Song, Lansheng Zhang, Dongsheng Pei

**Affiliations:** ^1^ Department of Pathology, Xuzhou Medical University, Xuzhou, China; ^2^ Department of Human Anatomy, Xuzhou Medical University, Xuzhou, China; ^3^ Department of Orthopedics, The Affiliated Suqian Hospital of Xuzhou Medical University, Suqian, China; ^4^ Department of Oncological Radiotherapy, The Second Affiliated Hospital of Xuzhou Medical University, Xuzhou, China

**Keywords:** lipid metabolism, CPT2, FAO, immune cell infiltration, immunotherapy

## Abstract

**Background:** Tumors frequently evade immune surveillance through multiple pathways to escape T cell recognition and destruction. Previous studies indicated that lipid metabolism alteration could affect the anti-tumor immunity of cancer cells. Nonetheless, the studies that investigated lipid metabolism-related gene for cancer immunotherapy are still few.

**Materials and methods:** By mining the TCGA database, we screened out carnitine palmitoyltransferase-2 (CPT2), a key enzyme in the fatty acid β-oxidation (FAO) process associated with anti-tumor immunity. We then analyzed the gene expression and clinicopathological features of CPT2 using open-source platforms and databases. Molecular proteins interacting with CPT2 were also identified using web interaction tools. Subsequently, the relationship between CPT2 and survival was analyzed in cancer patients.

**Results:** Our study revealed that CPT2 played a vital role in tumor microenvironment and immune response signaling pathways. We have also demonstrated that increased CPT2 gene expression could enhance the level of tumor immune cell infiltration. Furthermore, high CPT2 expression positively related with overall survival associated with immunotherapy. CPT2 expression was also associated with the prognosis of human cancers, suggesting that CPT2 may be a potential biomarker for predicting the efficacy of cancer immunotherapy.

**Conclusion:** To the best of our knowledge, the relationship between CPT2 and tumor immune microenvironment was first proposed in this study. Therefore, further studies on CPT2 may provide new insights into the development of effective cancer immunotherapy.

## Introduction

It is well established that immune escape and immune infiltration are associated with tumor growth and development (Alemohammad et al., 2022). Different types of cells such as T (Treg) cells, M2-type macrophages and immune checkpoints during the immune response are involved in the regulation of the immune regulatory system (Butt and Mills, 2014). Immune checkpoint therapy has led to major breakthroughs in the treatment of tumors previously considered to be highly lethal. Specifically, the presence of tumor-infiltrating lymphocytes (TILs), T cell (CD4^+^, CD8^+^, NK, γδ), macrophages, dendritic cells (DCs), B cells, mast cells, and neutrophils, as well as their localization, formation and density, can influence both the immune response and patient survival (Nossal, 1975; Fridman et al., 2012). Improving the survival of cancer patients through tumor immunotherapy has brought a new revolution in the field of oncology (Waldman et al., 2020). Tumor immunotherapy has become a popular and advanced treatment nowadays. However, it now appears that immunotherapy has reached a bottleneck. Only by better understanding how cancer cells evade immunotherapy can cancer be better treated (Cerezo and Rocchi, 2020; Waldman et al., 2020).

Cancer cells take up nutrients in tumor immune microenvironment (TIME) to create a favorable growing environment for tumor cells. Multiple immune cells and stromal cells make up the tumor tissue and they collaborate with each other to facilitate the reprogramming of the metabolic environment (Frades et al., 2021). A key determinant of the efficacy of immunotherapy appears to be metabolic interference between immune effector cells and tumor cells (Cerezo and Rocchi, 2020). The well-known Warburg effect is that cancer cells rely on glycolysis even in the presence of sufficient oxygen (Warburg, 1956; Li and Zhang, 2016). In addition, metabolic reprogramming of cancer cells also manifests as aberrant lipid metabolism and the dysregulation of amino acid metabolism (Li and Zhang, 2016). Although most of the knowledge about metabolic dysregulation in cancer has a focus on carbohydrates, we decided to focus our interest on the lipid metabolism of cancer cells (Merino Salvador et al., 2017; Cerezo and Rocchi, 2020). And the increase in *de novo* lipogenesis is considered to be a new hallmark of many aggressive forms of cancer (Robey et al., 2015). The metabolism of lipids is greatly altered in the proliferating cells. Unlike normal cells, the biosynthesis of fatty acids (FAs) in tumor tissue is significantly increased to meet the demand for local nutrients, oxygen, lipid synthesis of membrane and signaling molecules (Robey et al., 2015). Lipid metabolism dysfunction in the tumor microenvironment (TME) has been shown to affect tumor-associated immune responses (Yu et al., 2021). And a number of enzymes involved in lipid metabolism have been identified to be associated with carcinogenesis (Menendez and Lupu, 2007; Zhou et al., 2013; Mashimo et al., 2014; Bian et al., 2021).

Carnitine palmitoyltransferase (CPT) is an acyltransferase localised to the inner mitochondrial membrane in the process of fatty acid oxidation (FAO). Carnitine palmitoyltransferase (CPT) deficiency is a common disease that occurs during mitochondrial fatty acid oxidation. (Liu et al., 2022). The CPT system consists of two proteins, CPT1 and CPT2, which have different functions and localizations (Bonnefont et al., 2004). CPT1 locates the external mitochondrial membrane and acts as a catalyst for the formation of acylcarnitins based on carnitine and acyl CoA. The main function of inner mitochondrial membrane CPT2 is to form acyl coenzyme A from acylcarnitine and CoA (Bonnefont et al., 2004). In recent years, the role of the CPT system in cancer metabolic mechanisms has been updated due to the increasing understanding of fatty acid oxidation in cancer (Li et al., 2021). Studies have shown that PD-1 supports lipolysis by upregulating CPT1A and FAO (Patsoukis et al., 2015). Moreover, targeted CPT1 therapy reduces the formation of invasive pseudopodia in hepatocellular cancer cells and eliminates the tumor-promoting effect of tumor-associated macrophages (TAMs) (Su et al., 2020). CPT2 deficiency or dysfunction usually results in some lipid metabolic disorders such as diabetes, cardiac hypertrophy, NAFLD and obesity (Gu et al., 2017; Pereyra et al., 2020). CPT2 is highly expressed in recurrent breast cancer and associated with poor prognosis of patients. CPT2 increase promotes the growth and radiation resistance of breast cancer cells (Han et al., 2019). However, CPT2 was shown to be downregulated in HCC tissues and associated with vascular invasion and tumor tissue differentiation (Fujiwara et al., 2018; Lin et al., 2018). Silencing of CPT2 contributes to the metastatic and tumorigenic activity of hepatocellular carcinoma cells (Lin et al., 2018). Similarly, CPT2 knockdown has been demonstrated to promote the proliferation, migration and invasion of CRC cells *in vitro*, and to induce cell cycle arrest and apoptosis (Li et al., 2021). In addition, upregulation of CPT2 has been shown to attenuate CRC tumor development and increase chemosensitivity via a hyperactive Wnt/β-catenin pathway *in vivo* (Liu et al., 2022). Unfortunately, the role of CPT2 in tumor immune microenvironment remains unknown.

**FIGURE 1:**
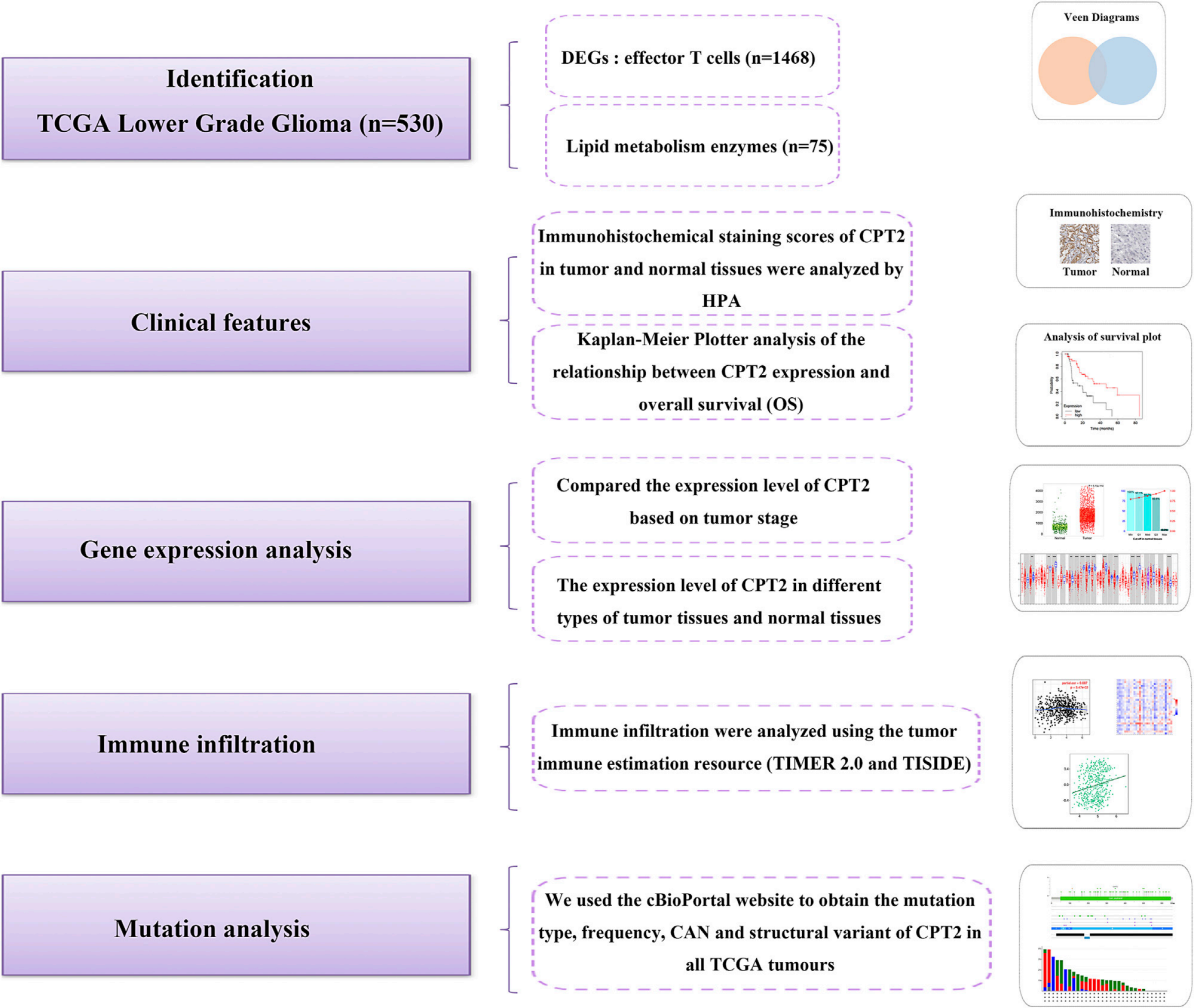
The analytical process of exploring the function of CPT2 in the pan-cancer landscape.

In this study, we explored the expression of CPT2 in human cancer and identified its relationship with the level of immune cell infiltration. And the entire process of exploring the function of CPT2 in the pan-cancer landscape was showed in Figure 1.

## Methods

### Enrichment analysis of CPT2-related genes

TCGA database were used to screen lipid metabolism enzymes related to effector T cells. The screened genes were then plotted via a Venn diagram using the JVENN (http://jvenn.toulouse.inra.fr/app/example.html) website. Through the utilization of the purity-adjusted Spearman’s rank correlation test, *p*-values and partial cor values were obtained. STRING is a database dedicated to the study of protein interactions throughout the organism (Huang et al., 2018). We submitted CPT2 on the STRING (https://string-db.org) website. STRING generated a network of key proteins that have interactions with CPT2. BioGRID (Biological General Repository for Interaction Datasets, thebiogrid.org) is a comprehensive, open access biomedical database resource that collates protein, genetic and chemical interactions from multiple species including yeast, worms, flies, mice and humans. We searched for CPT2 on BioGRID (https://thebiogrid.org) website and then obtained a genes network associated with CPT2 from the same organism and different organism.

### Linkedomics database

The open multi-omics database LinkedOmics (http://www.linkedomics.org/login.php) contains data from 10 cancer cohorts from the Clinical Proteomics Tumor Analysis Consortium (CPTAC) and 32 TCGA cancer types. The gene set enrichment analysis (GSEA) and GO analysis (biological process) of CPT2 were generated from LinkedOmics.

### Differential expression analysis

The Human Protein Atlas (HPA) (https://www.proteinatlas.org) provides information on the tissue and cellular distribution of 26,000 human proteins. HPA covers the expression of each protein in 64 cell lines, 48 human normal tissues and 20 tumor tissues. We used the HPA database to evaluate immunohistochemical staining of CPT2 in liver cancer, stomach cancer, lung cancer, renal cancer, pancreatic cancer, colorectal cancer and normal tissue.

The Timer website’s DiffExp module displays the difference gene expression patterns in all TCGA tumors between the tumor and normal tissue. In a box plot, the distribution of gene expression levels is displayed, and the Wilcoxon test determines the statistical significance of differential expression. We analyzed whether CPT2 was up- or downregulated in tumors for each cancer type compared to normal tissues.

### TNMplot: Differential gene expression analysis

TNM plot (https://tnmplot.com/analysis/) is a comprehensive database using existing transcriptome-level datasets, which can be mined by comparing normal, tumor and metastasis data for all genes in real time. The entire database contains 56,938 samples, including 33,520 samples from 3,180 gene chip-based studies (453 metastatic, 29,376 tumorigenic and 3,691 normal samples), 11,010 samples from TCGA (394 metastatic, 9,886 tumorigenic and 730 normal samples), 1,193 samples from TARGET (1 metastatic, 1,180 tumorigenic and 12 normal samples) and 11,215 normal samples from GTEx. The Normal and Tumor analysis page provides detailed analysis for CPT2 in a selected tissue type using gene chip based data.

### Immune infiltration analysis

A thorough investigation of the relationship between cancerous cells and the host immune system is necessary in light of the recent clinical success of cancer immunotherapy in a range of tumor types. TIMER (https://cistrome.shinyapps.io/timer/) provides 6 major analytic modules that allow users to interactively explore the associations between immune infiltrates and a wide spectrum of factors, including gene expression, clinical outcomes, somatic mutations, somatic copy number alterations, differential expression and correlation analysis. TIMER applies the deconvolution algorithm to estimate the number of six different immune infiltrating cell types (B cells, CD4^+^ T cells, CD8^+^ T cells, neutrophils, macrophages, and dendritic cells). TISIDB (http://cis.hku.hk/TISIDB) is a web portal for studying function of specific genes in tumor-immune interactions through literature mining and high-throughput data analysis. Correlation of CPT2 and immune effector cells in Low grade glioma (LGG) was analyzed by TISIDB website.

### Survival analysis

The main objective of the Kaplan Meier plotter is to assess the correlation between expression of genes (more than 30,000 samples from 21 tumor types) and survival rates. Sources of the database include GEO, EGA and TCGA. One of the finest methods for calculating the percentage of participants who continue to live following therapy is the Kaplan-Meier estimate. The number of participants who survive or are saved following an intervention is measured over time in a clinical study or community trial to determine the intervention’s effectiveness. The study of cohort data is known as survival analysis, and survival time is the period of time between a stated point and the occurrence of an event, such as death (Goel et al., 2010). Survival curves were calculated using the Kaplan-Meier method, and differences between groups were compared using the log-rank test (Zou et al., 2021). *p*-values less than 0.05 were considered as a significant difference. We have not only investigated the prognosis of CPT2 in patients with liver, lung, kidney, thyroid, pancreatic and gastric cancers, but also analyzed the clinical association between immune characteristics and ultimate patient survival by Kaplan Meier.

### Genetic alteration analysis

The cBioPortal for Cancer Genomics is an online tool for exploring, visualizing, and interpreting multimodal cancer genomes data (http://cbioportal.org). The portal transforms genetic, epigenetic, gene expression, and proteomic processes into plainly understandable terms using molecular profiling data from cancer tissues and cell types. We obtained the frequency, mutation type, copy number alteration (CNA) and structural variants of TCGA tumors in the “Cancer Type Summary” by a quick search of CPT2 on the cBioPortal (https://www.cbioportal.org/) website. We obtained the mutation site information and 3D structure of CPT2 protein by “mutation” model.

### Proportion of immune cells analysis

Cancer Single-cell Expression Map (https://ngdc.cncb.ac.cn/cancerscem/index) is a freely accessible database for the gathering, examination, and presentation of single-cell RNA-Seq data from human cancer. With a thorough online analysis platform built into the database, a multi-level study sheds light on the tumor microenvironment of many types of human cancers.

### Gene-metabolite interaction analysis

Metabolic Atlas is a web platform for exploring and analyzing open-source genome scale metabolic models (GEMs). The purpose is to collect curated GEMs and align these models with FAIR principles. The website features GEM visualizations and comparisons, as well as access to resources, algorithms, other databases, and more general software applications. Metabolic Atlas is designed for use in metabolomics, clinical chemistry, biomarker development, and general education.

## Results

### Identification of CPT2 as the hub gene

**FIGURE 2:**
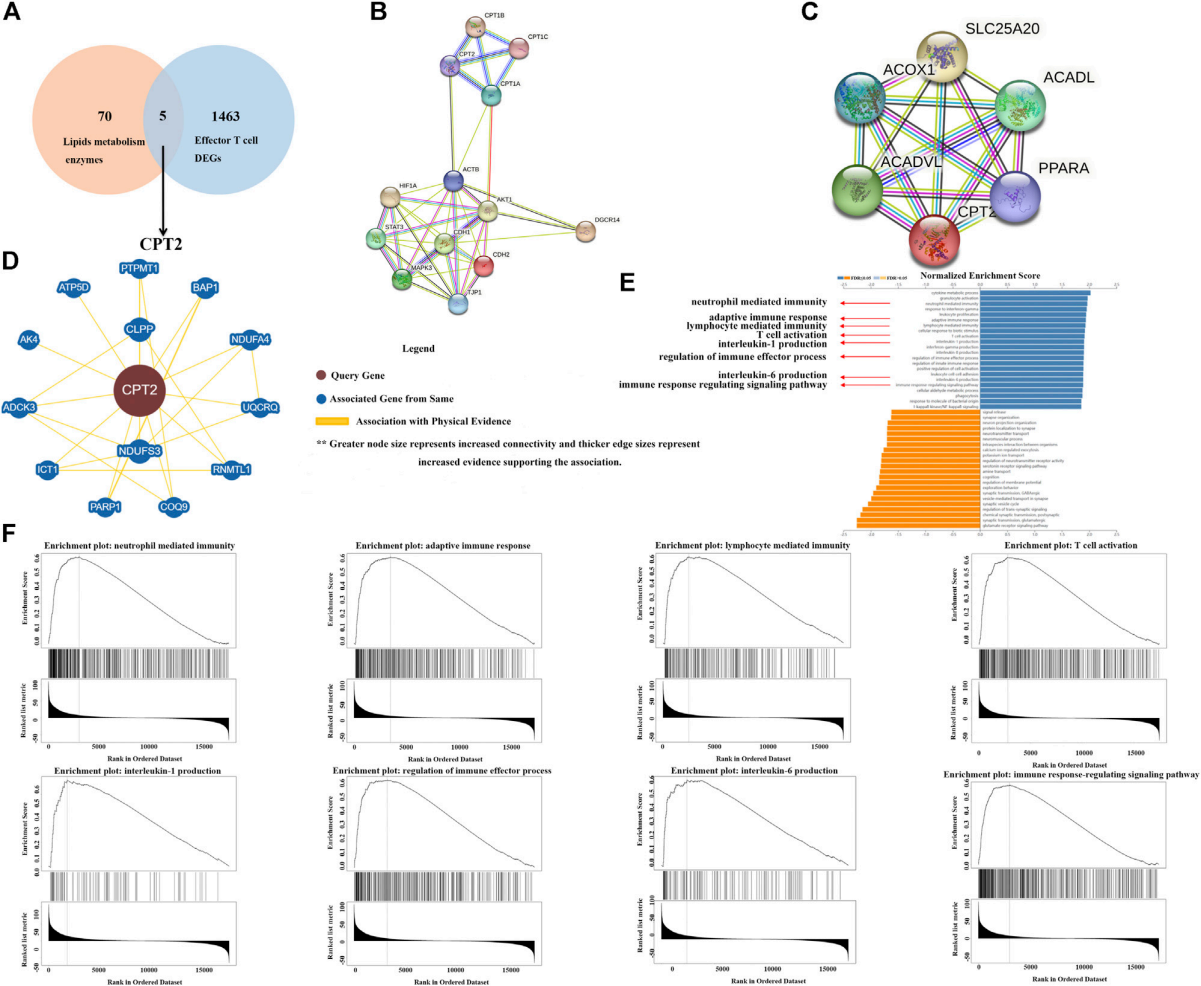
Identification of CPT2 as the hub gene. **(A)** Venn diagram displaying the overlaps of top 75 enzymes involved in lipid metabolism and Genes with a significantly positive correlation with Effector T cell DEGs. **(B, C)** Two alternative maps of the CPT2 protein interaction network using the two search methods available on the string website: single protein by name/identifier and geneset by pathway/process/disease/publication. **(D)** Examining significant CPT2-related protein interactions in the BioGRID database. **(E, F)** GO biological process analysis and GESA analysis of CPT2 in LGG.

To explore the relationship between lipid metabolism and tumor immunity, we screen out remarkable lipid metabolism-related genes associated with effector T cells through TCGA database. In total, 1,468 differentially expressed genes (DEGs) related to effector T cells were obtained. Among them, there are five lipid metabolic enzymes (LPCAT2, PLCG2, PLCB2, CPT2 and PLA2G4A) that are significantly associated with effector T cells (Figure 2A). The two types of searches on the string website, single protein by name/identifier and geneset by pathway/process/disease/publication, allow us to obtain two different maps of the CPT2 protein interaction network (Figure 2B, C). Through BioGRID websites, we were able to get the PPI network to analyze the CPT2 protein interactions (Figure 2D). Moreover, to understand the biological function of CPT2 in LGG, GO functional enrichment analysis revealed that CPT2 was largely associated with immune regulation including neutrophil mediated immunity, adaptive immune response, lymphocyte mediated immunity, T cell activation, interleukin-1 production, regulation of immune effector process, interleukin-6 production and immune response regulating signaling pathway (Figure 2E). More specifically, gene set enrichment analysis (GSEA) demonstrated a positive correlation between CPT2 expression levels and immune regulation including neutrophil mediated immunity, adaptive immune response, lymphocyte mediated immunity, T cell activation, interleukin-1 production, regulation of immune effector process, interleukin-6 production and immune response regulating signaling pathway (Figure 2F).

### Analysis of CPT2 and clinical features in human cancer

**FIGURE 3:**
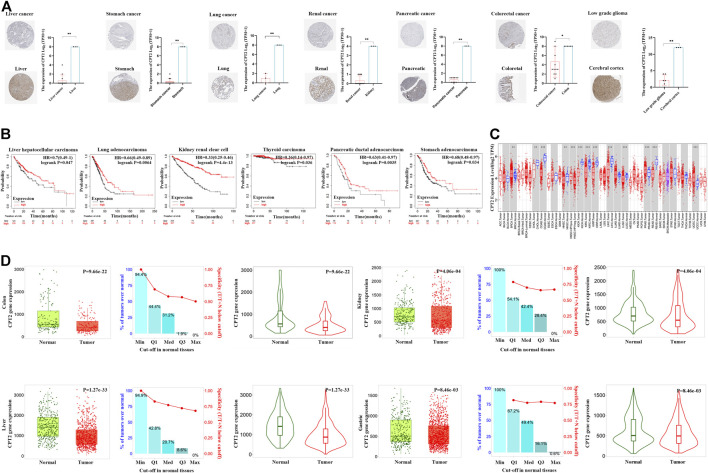
Analysis of CPT2 and clinical features in human cancer. **(A)** CPT2 protein expression levels in normal and tumor tissues were compared through immunohistochemical images involving liver cancer, stomach cancer, lung cancer, kidney cancer, pancreatic cancer, colorectal cancer, and low grade glioma. **(B)** Survival curves for patients with high (red) and low (black) expression of CPT2 were displayed using the Kaplan-Meier Plotter database for liver hepatocallular carcinoma, lung adenocarcinoma, kidney renal clear cell thyroid carcinoma, pancreatic ductal adenocarcinoma, and stomach adenocarcinoma. The threshold of Cox *p*-value <0.05. **(C)** The expression level of CPT2 in different types of tumor tissues and normal tissues in TIMER database. Distributions of gene expression levels are displayed using box plots. Red boxes represent tumor tissues; blue boxes represent normal tissues. The Wilcoxon test results’ statistical significance is indicated by the number of stars (∗: p−value < 0.05 ; ∗∗: p−value <0.01 ; ∗∗∗: p−value <0.001 ). As displayed in gray columns when normal data are available. **(D)** TNMplot database data concerning the expression pattern of CPT2 in human cancer. Red boxes represent tumor tissues; green boxes represent normal tissues. ∗ P < 0.05 , ∗∗ P < 0.01 .

To assess the clinical features of CPT2 expression, we evaluated the immunohistochemical staining of CPT2 via HPA database. Results indicated that CPT2 was strongly marked in normal tissues than tumor tissues (Figure 3A). In liver hepatocellular Carcinoma (LIHC), lung adenocarcinoma (LUAD), kidney renal clear cell carcinoma (KIRC), thyroid carcinoma (THCA), pancreatic adenocarcinoma (PAAD), and stomach adenocarcinoma (STAD), low levels of CPT2 expression were found to be related with a poor overall survival (OS), according to an investigation of the Kaplan-Meier Plottr database. (Figure 3B). Subsequently, we next used the TIMER website to examine the differential expression of CPT2 in various tumor tissues and normal tissues. The findings demonstrated that CPT2 expression was significantly lower in cholangiocarcinoma (CHOL), colon adenocarcinoma (COAD), head-neck squamous cell carcinoma (HNSC), kidney chromophobe (KICH), kidney renal papillary cell carcinoma (KIRP), LIHC, lung squamous cell carcinoma (LUSC) and rectal adenocarcinoma (READ) (Figure 3C). Furthermore, it is identified that CPT2 was overexpressed in normal tissues compared with tumor tissues by TNMplot analysis (Figure 3D).

### CPT2 is associated with immune cells in the tumor microenvironment of LGG

**FIGURE 4:**
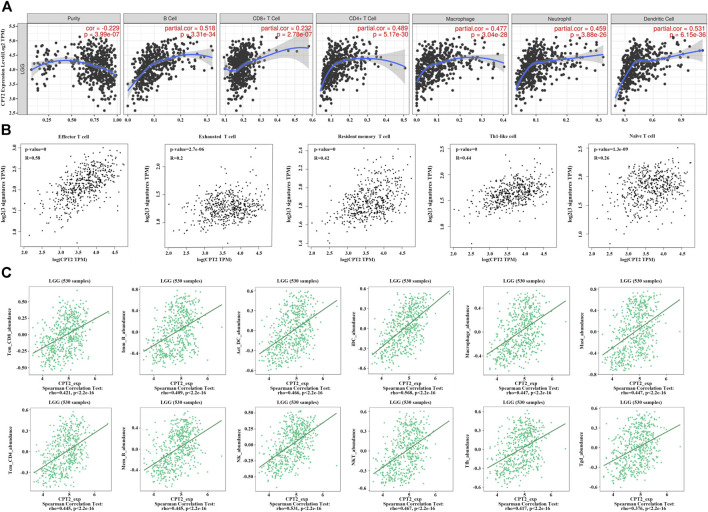
Correlations of CPT2 expression with immune cells. **(A)** Correlation between CPT2 expression and B cell, CD8^+^ T cell, CD4^+^ T cell, Macrophage, Neutrophil and Dentritic cell in LGG. Scatter plots display purity-corrected partial Spearman’s rho values as well as statistical significance. The gene expression levels against tumor purity are always displayed on the left-most panel. **(B)** Correlation between CPT2 expression and highly connected immune cell, including Effector T cell, Exhausted T cell, Resident memory T cell Th1-like cell and Naïve T cell in LGG. **(C)** Bioinformatics analysis of the correlation between CPT2 and immune effector cells using TISIDB database.

It is well established that cells of the immune system in the tumor microenvironment may have an impact on the prognosis and survival of cancer patients. Based on the TIMER 2.0 database and taking into account the strong connection between CPT2 and immune infiltration, we discovered a substantial positive correlation between CPT2 and the degree of immune cell infiltration in LGG. (Figure 4A). Furthermore, we discovered a positive correlation between CPT2 expression and the infiltration levels of effector T cells, exhausted T cells, resident memory T cells, Th1-like cells, and naive T cells (Figure 4B). The correlations of immune cell infiltrations and CPT2 in LGG were systematically investigated through TISIDB database, which revealed significant positive correlations between CPT2 expression and Tcm CD8 T-cell subsets, Imm B-cell, Act DC-cell, iDC-cell, Macrophage cell, Mast cell, Tcm CD4 T-cell, Mem B-cell, NK cell, NK T-cell, Tfh cell and Tgd cell (Figure 4C). These results indicated that CPT2 was positively correlated with immune cells in tumor microenvironment.

### Correlation between CPT2 expression and overall survival associated with immunotherapy

**FIGURE 5:**
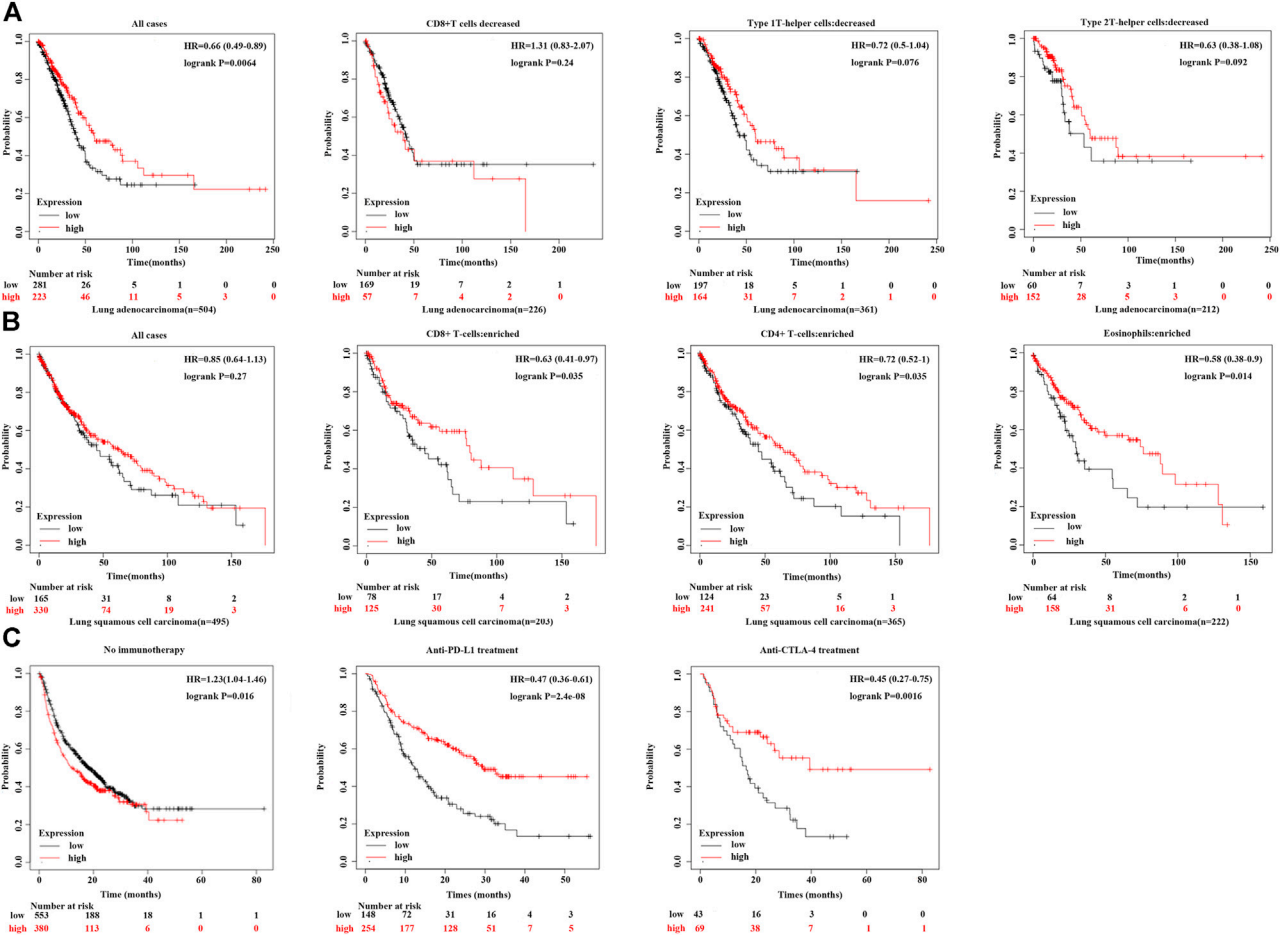
Correlation between CPT2 expression and overall survival associated with immunotherapy. **(A, B)** Kaplan-Meier curves show the impact of CPT2 and immune cells (CD4^+^ T cells, CD8^+^ T cells, type 1 T-helper cells, type 2 T-helper cells) expression levels on patient survival prognosis. **(C)** Survival analysis of all tumor types in Kaplan-Meier include Bladder, Esophageal adeno, Glioblastoma, Hepatocellular carcinoma, HNSCC, Melanoma, NSCLC, NSLC, Urothelial treated with immune checkpoint (PD-L1 and CTLA-4) or without any immunotherapy.

Figure 5A illustrates that a reduction in CD8^+^ T cells and type 1 T-helper cells decreases survival in lung adenocarcinoma patients with high CPT2 expression. Moreover, Figure 5B indicates that overall survival was significantly higher when CD8^+^ T cells, CD4^+^ T cells and eosinophils were enriched in squamous cell lung cancer patients with high CPT2 expression**.** In order to learn more about the connection between CPT2 and immunotherapy, we discovered that patients with lung squamous cell carcinoma who received anti-PDL1 and anti-CTLA-4 treatment had higher survival rates when CPT2 was highly expressed (Figure 5C). These results confirmed the positive relationship between CPT2 expression and overall survival associated with immunotherapy.

### CPT2 positively regulates immune cells in LGG

**FIGURE 6:**
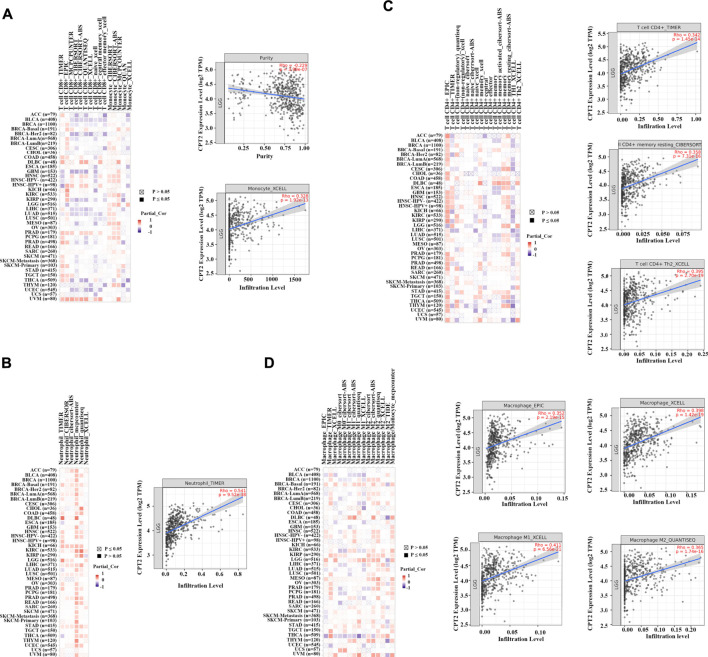
Heat maps and scatter plots of CPT2 correlated with immune cell infiltration level. The left panels are heat maps showing the correlation between all tumor types and immune cells in TCGA, with red representing positive correlation and blue representing negative correlation, the darker the color the higher the correlation. The right panels are scatter plot. **(A)** The relationship between CPT2 expression and CD8^+^ T cells, and monocytes infiltration levels was analyzed by the XCELL algorithm in LGG. **(B)** The relationship between CPT2 expression and neutrophil infiltration level was analyzed by the TIMER algorithm in LGG. **(C)** The relationship between CPT2 expression and CD4^+^ T cells infiltration level was analyzed by the CIBERSORT-ABS and XCELL algorithms in LGG. **(D)** The relationship between CPT2 expression and Macrophage infiltration level was analyzed by the EPIC, XCELL, and QUANTISEQ algorithms in LGG.

TIMER 2.0 web server was applied to assess the effects of CPT2 on the level of immune cell infiltration in pan-cancer. It was demonstrated that CPT2 promoted the infiltration of CD8^+^ T cells and monocytes in LGG (Figure 6A); CPT2 promoted the infiltration of neutrophil in LGG (Figure 6B); CPT2 promoted the infiltration of CD4^+^ T cell in LGG (Figure 6C); CPT2 promoted the infiltration of Macrophage in LGG (Figure 6D). These data demonstrated that CPT2 might positively regulate immune cells in LGG.

### Mutation analysis of CPT2 in human cancer

**FIGURE 7:**
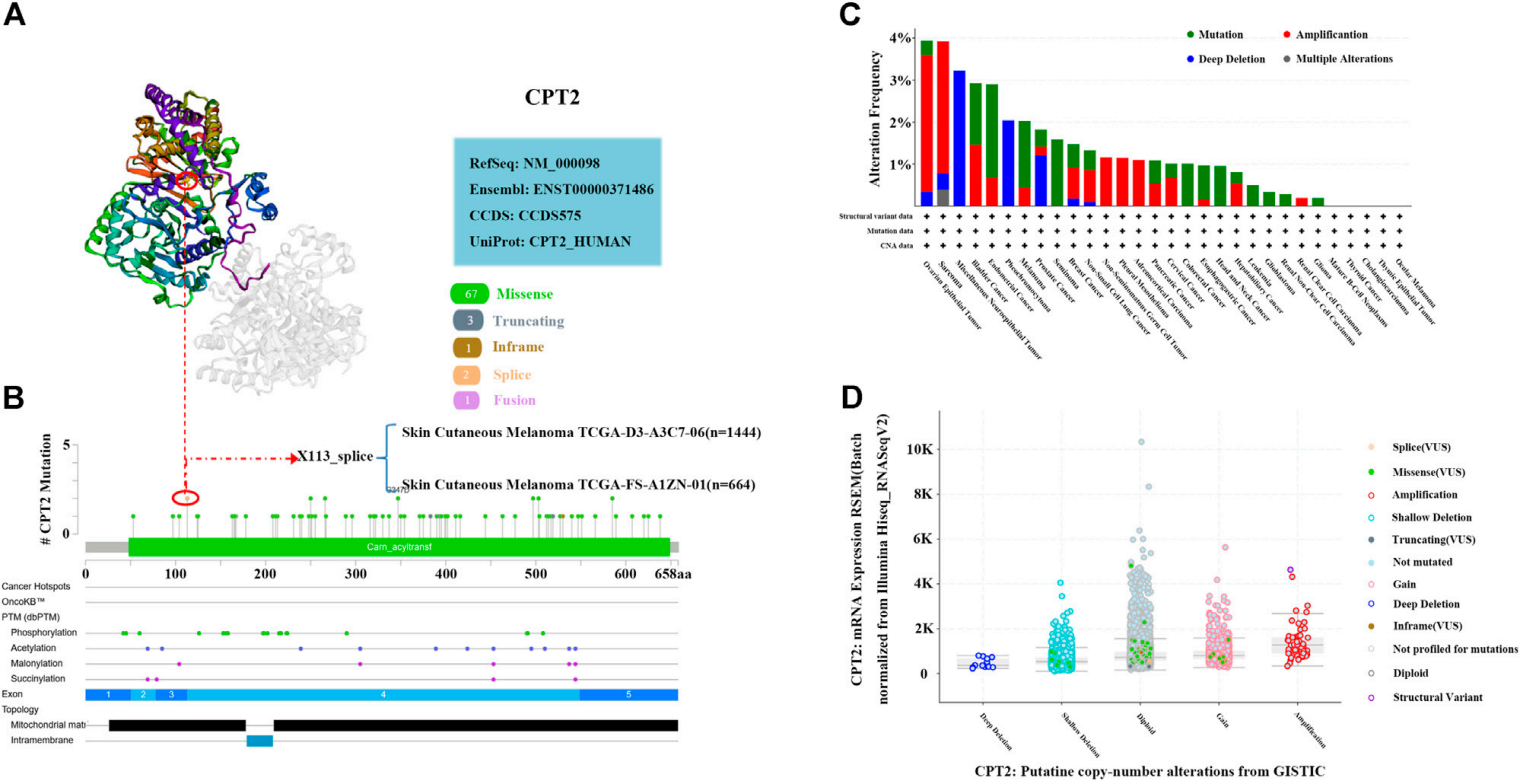
Mutation analysis of CPT2 in human cancer. **(A)** X113_splice mutation site of CPT2 in the 3D structure at cBioPortal website. **(B)** A total of 74 mutation sites were detected between amino acids 0 and 658 of CPT2 protein at cBioPortal website. **(C)** Alteration frequency of CPT2 in different tumor types. **(D)** A plot showing the relationship between CPT2 mRNA abundance and CNA in the CPT2 gene in tumors from the selected pan-cancer atlas study.

Figure 7A illustrated the X113_splice altered site of 3D structure of CPT2 protein. An alteration at position 113 of the CPT2 protein has been found in cutaneous melanoma (X113_splice). According to the mutation sites and types, we found missense mutation of CPT2 as the main mutation type (Figure 7B). The mutation type, frequency, CAN, and structural variant of CPT2 in all TCGA cancers were obtained from the cBioPortal website. According to Figure 7C, ovarian epithelial tumors had the greatest modification frequency of any TCGA tumors (3.94%), with amplification accounting for the majority of the frequency (3.25%). Analysis of CPT2 in pan-cancer illustrated that CPT2 is amplified in a subset. The cBioPortal results demonstrate that the samples with amplified CPT2 have higher levels of CPT2 mRNA. (Figure 7D).

## Discussion

Regulatory B cells, CD4^+^ Th2 cells, bone marrow myeloid-derived suppressor cells (BM-MDSCs), CD4^+^ T regulatory cells suppressor cells and alternatively activated macrophages (M2) are engulfed by the tumor development (Edechi et al., 2019). The findings suggested that CPT-1A expression and methylation is negatively associated with these cells, suggesting that survival and prognosis may be affected by inhibiting tumor-infiltrating lymphocytes (TILs) response (Das et al., 2022). In an earlier study, CPT-1A was shown to be a novel biomarker for the diagnosis and prediction of breast cancer (Rhodes et al., 2004). CPT-1A genomic alterations have been detected, in 9% of breast cancers. Alterations in the CPT1A genome have been confirmed to be associated with poor prognosis. Moreover, CPT-1A reduces the expression of CPT-1A through methylation and may be associated with a good prognosis. To date, numerous studies have demonstrated that CPT1 is closely associated with the occurrence and development of tumor cells in lung cancer (Zaugg et al., 2011), colorectal cancer (Wang et al., 2018), breast cancer (Linher-Melville et al., 2011), hepatocellular carcinoma (Brown et al., 2018), gastric cancer (Wang et al., 2020), ovarian cancer (Shao et al., 2016) and prostate cancer (Joshi et al., 2019). Nevertheless, the impact of CPT2 on the progress of cancers is still not well understood.

To better understand the role of CPT2 in cancer development and prognosis, we performed comparative data mining on numerous gene expression data sets. In the current study, we investigated the impact of CPT2 on pan cancer and the resulting clinical implications. We first performed differential and survival analysis based on TCGA and GEO databases. We found that CPT2 was highly expressed in most normal tissues compared to cancers, and its low expression was associated with poor prognosis in LGG. A total of 40 CPT2 mutations have been identified, distributed throughout the coding sequence of the gene. In addition to the prevalent S113L exon 3, exons 4 and 5 were hotspots for mutations. Missense mutations were the predominant mutation type, accounting for about 77%, while the remaining 23% were predicted to be truncated proteins (Bonnefont et al., 2004). Through the mutation analysis website, we found that CPT2 had a high mutation rate in ovarian epithelial tumors and sarcoma, and the mutation types were predominantly missense mutations.

Fatty acids are a large, critical, energy source that can meet the substantial fuel needs of acutely propagating cancer cells (De Rosa et al., 2012; Hoang-Minh et al., 2018; Duman et al., 2019). Recently, two groups have further revealed that FA metabolism accelerates the development of brain metastases from breast cancer (Gupta et al., 2020), while glioblastoma multiforme (GBM) metabolism is considerably less dependent on glycolysis (Reinfeld et al., 2021). Moreover, it has been reported that FAs provide essential nutrients for the growth of tumor cells, and that a deficiency of FAs can lead directly to the death of FA-dependent tumor cells (Aloia et al., 2019). Previously, many studies have demonstrated regulation of FA synthesis in immune cells is also essential for immunity against cancer and the synthesis of FA is regulated by the metabolism of lipids and lipoproteins through the PPAR activators (Nakamura et al., 2014; Qian et al., 2018). However, it is unclear whether enhanced FA metabolism in recurrent and radioresistant GBM, can generate or promote a state of immunosuppression leading to immune evasion (Jiang et al., 2022). FA deficiency also blocks the growth of MDSC and Tregs that use FAOs. Promoting the FOA process may be an effective strategy to improve antitumor immunity, as FAO has been suggested to favour the longevity of memory T cells (Chowdhury et al., 2018). Furthermore, several lipid metabolism-related genes were identified to be associated with tumor immunology. Inhibition of ZDHHC9, a palmitoyl transferase, could interrupt the palmitoylation of PD-L1, sensitized tumor cells to immune T cell attacking (Yang et al., 2019; Liao et al., 2021). The palmitoyltransferase ZDHHC3 (DHHC3) has also been shown to be involved in acetyl transfer during PD-L1 palmitoylation (Yao et al., 2019; Liao et al., 2021). PD-L1 palmitoylation can inhibit PD-L1 expression and function to promote cancer cell killing by T cells (Liao et al., 2021). Accumulating studies strengthened the role of lipid metabolism enzymes for TIME modulation in tumor expansion (Hodson and Fielding, 2013; Bai et al., 2015; Cotte et al., 2018).

Hence, our study revealed that CPT2, a key enzyme in the FAO process, was positively associated with the level of immune cell infiltration, which suggested that CPT2 could affect tumor progression by reprogramming lipid metabolism in the TIME. Based on previous reports of PD-1 inducing upregulation of CPT1A and FAO (Patsoukis et al., 2015), we further explored the relationship between CPT2 and immunotherapy. It was demonstrated that survival in squamous cell lung cancer was significantly increased when CPT2 was highly expressed and treated with anti-PDL1 or anti-CTLA-4. Therefore, further studies would be warranted to verify the value of the prognostic and predictive benefit of CPT2 agonist.

In addition, there is increasing evidence that tumor metabolism can affect immunological molecules by releasing metabolites as well as to being essential for supporting growth and survival. CPT2, a key molecule in lipid metabolism, and the crosstalk between tumor metabolites including Malonyl-CoA, 1,1-Dimethylbiguanide, L-Palmitoylcarnitine, Estradiol, Propionyl-CoA, Palmityl-CoA, Palmitaldehyde, Coenzyme A, and Palmitic acid may offer new hope for tumor immunotherapy.

## Data Availability

The datasets presented in this study can be found in online repositories. The names of the repository/repositories and accession number(s) can be found in the article/Supplementary Material.
