# Supplementary figures and images for: Identification of a lipid metabolism-related gene for cancer immunotherapy
Source: Front Pharmacol. 2023 May 12;14:1186064. doi: 10.3389/fphar.2023.1186064 (PMC10213444; doi:10.3389/fphar.2023.1186064)

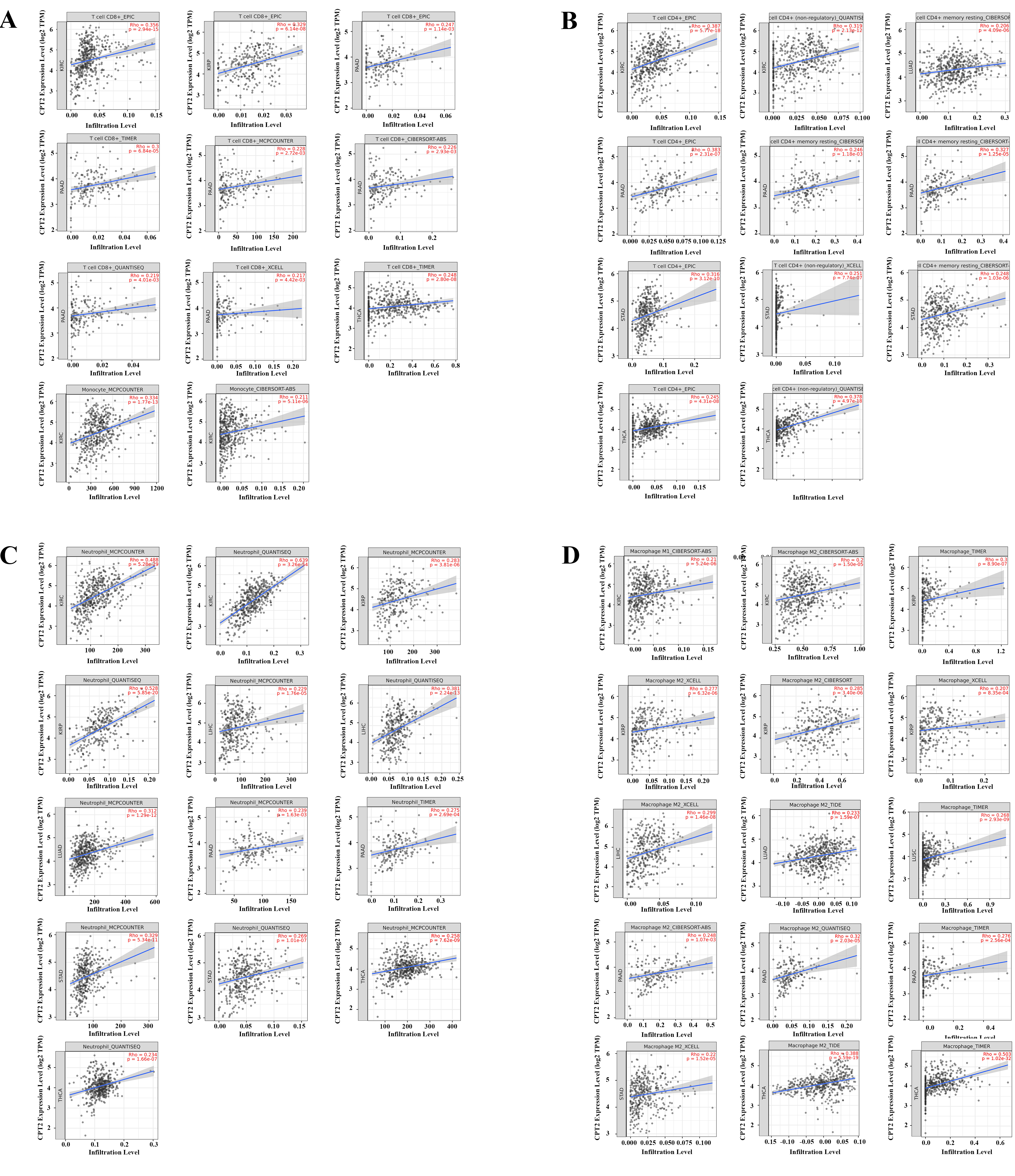

Supplement: Supplementary file 2 [file Image3.TIF]

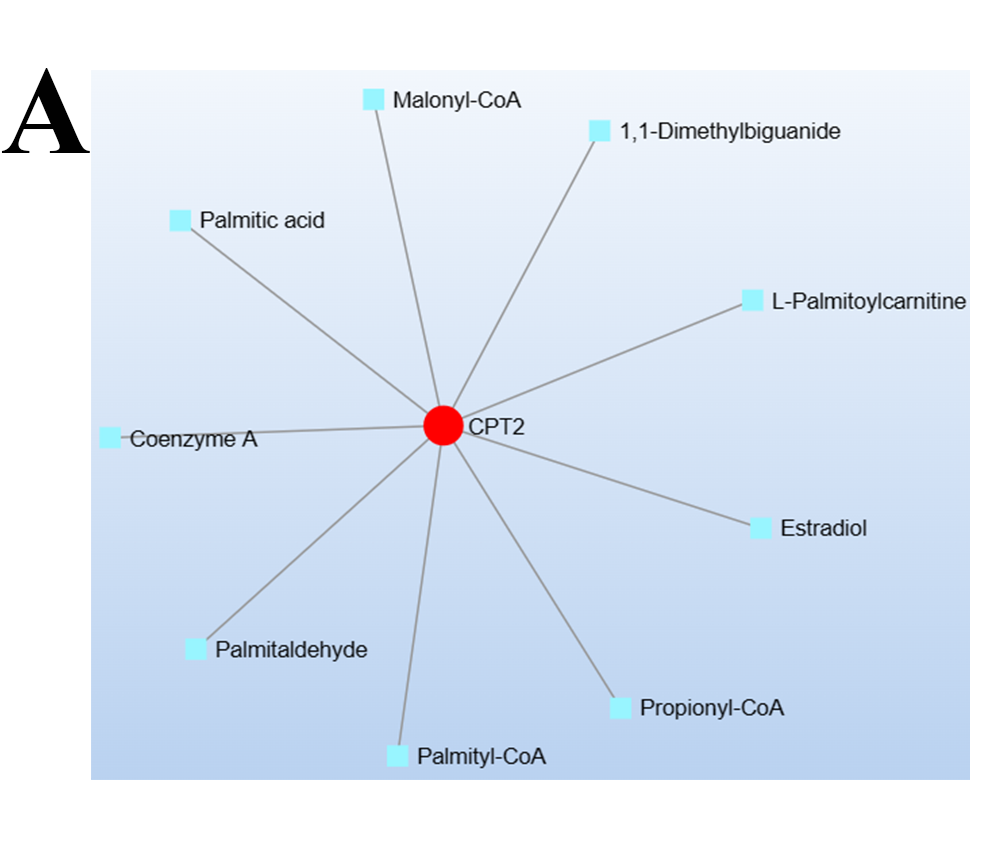

Supplement: Supplementary file 3 [file Image2.TIF]

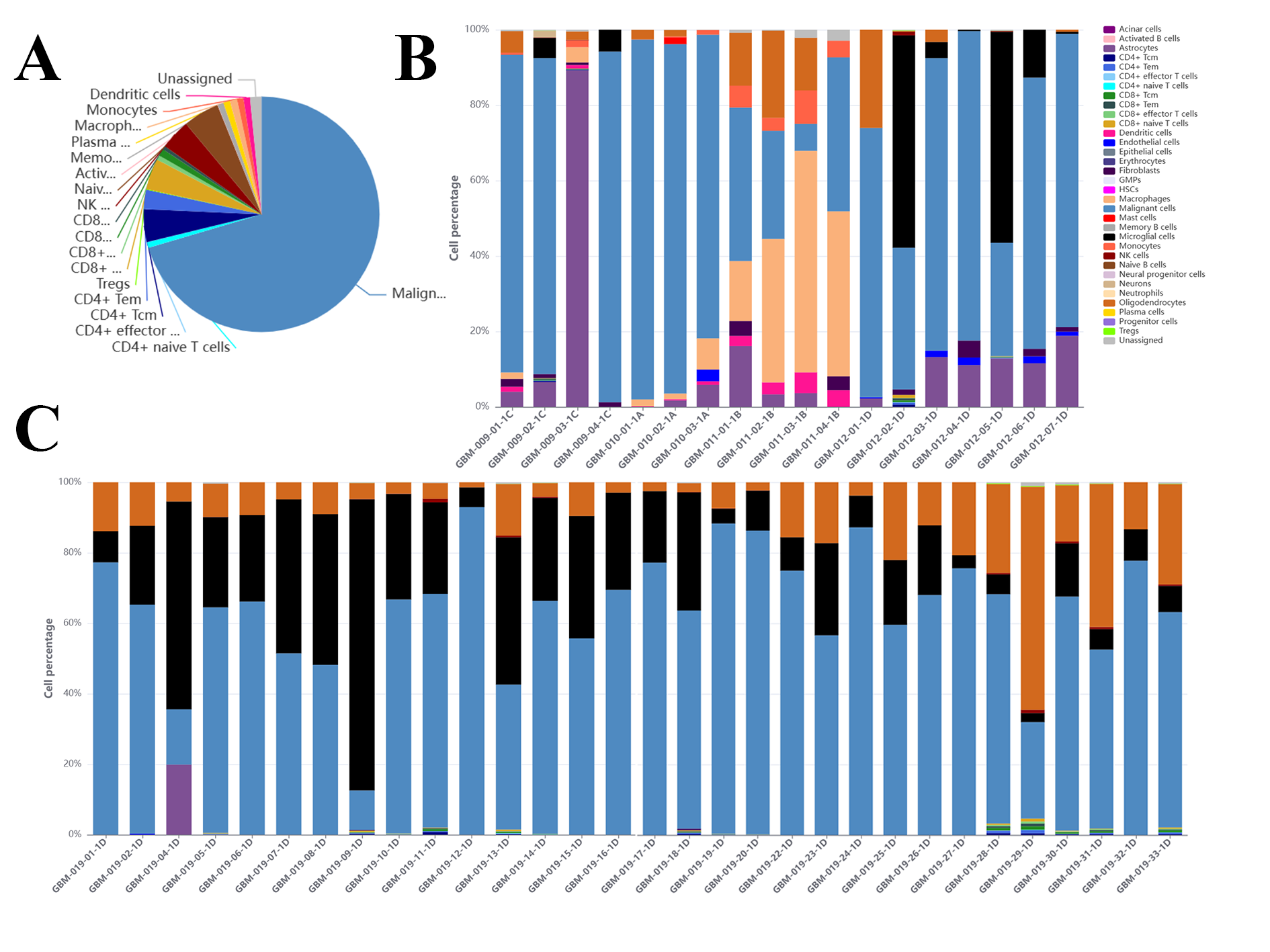

Supplement: Supplementary file 4 [file Image1.TIF]
